# Molecular Biology and Evolution of Cancer: From Discovery to Action

**DOI:** 10.1093/molbev/msz242

**Published:** 2019-10-23

**Authors:** Jason A Somarelli, Heather Gardner, Vincent L Cannataro, Ella F Gunady, Amy M Boddy, Norman A Johnson, Jeffrey Nicholas Fisk, Stephen G Gaffney, Jeffrey H Chuang, Sheng Li, Francesca D Ciccarelli, Anna R Panchenko, Kate Megquier, Sudhir Kumar, Alex Dornburg, James DeGregori, Jeffrey P Townsend

**Affiliations:** 1 Department of Medicine, Duke University Medical Center, Durham, NC; 2 Duke Cancer Institute, Duke University Medical Center, Durham, NC; 3 Sackler School of Graduate Biomedical Sciences, Tufts University, Medford, MA; 4 Department of Biology, Emmanuel College, Boston, MA; 5 Department of Anthropology, University of California, Santa Barbara, CA; 6 Department of Biology, University of Massachusetts, Amherst, MA; 7 Department of Biostatistics, Yale School of Public Health, New Haven, CT; 8 The Jackson Laboratory for Genomic Medicine, Farmington, CT; 9 Cancer Systems Biology Laboratory, The Francis Crick Institute, London, United Kingdom; 10 King’s College London, London, United Kingdom; 11 Department of Pathology and Molecular Medicine, School of Medicine, Queen’s University, Kingston, ON, Canada; 12 Ontario Institute of Cancer Research, Toronto, ON, Canada; 13 Broad Institute, Massachusettes Institute of Technology and Harvard University; 14 Institute for Genomics and Evolutionary Medicine, and Department of Biology, Temple University, Philadelphia, PA; 15 North Carolina Museum of Natural Sciences, Raleigh, NC; 16 Department of Biochemistry and Molecular Genetics, University of Colorado Anschutz Medical Campus, Aurora, CO; 17 Department of Ecology and Evolutionary Biology, Yale University, New Haven, CT; 18 Program in Computational Biology and Bioinformatics, Yale University, New Haven, CT

**Keywords:** cancer, fitness landscapes, metastasis, genomics, tumor phylogenetics, comparative oncology

## Abstract

Cancer progression is an evolutionary process. During this process, evolving cancer cell populations encounter restrictive ecological niches within the body, such as the primary tumor, circulatory system, and diverse metastatic sites. Efforts to prevent or delay cancer evolution—and progression—require a deep understanding of the underlying molecular evolutionary processes. Herein we discuss a suite of concepts and tools from evolutionary and ecological theory that can inform cancer biology in new and meaningful ways. We also highlight current challenges to applying these concepts, and propose ways in which incorporating these concepts could identify new therapeutic modes and vulnerabilities in cancer.

The vast majority of cancer-related deaths occur in the context of metastatic spread of therapy-resistant cell lineages; and the progression from normal tissue to a localized, treatment-responsive, metastatic, and therapy-resistant disease is fundamentally an evolutionary process (Nowell 1976). During this process a diverse population of cancer cells is subject to selective forces encountered within the tissue ecology of the body. Restrictions on space (Chkhaidze et al. 2019), nutrients (Lyssiotis and Kimmelman 2017), oxygen (Amend and Pienta 2015), and other microenvironmental factors all select on clonal molecular variants within the primary tumor. These microenvironmental conditions can also induce a migratory and invasive phenotype that promotes tumor cell dissemination (Jung et al. 2015; Jolly et al. 2017) and subsequent metastatic diversification in novel environments (reviewed in Labelle and Hynes 2012). In addition to the environments encountered within the primary and metastatic niche, therapy also imposes an intense selective pressure on cancer cells, sometimes focused on individual genes or gene domains, and often leads to rapid emergence of therapy-resistant subclones (Enriquez-Navas et al. 2016). 

While the population diversity subject to selective forces is most often associated with genetic diversity (Ku et al. 2017; Mu et al. 2017), other factors also can create phenotypic diversity within a cancer cell population. These factors include DNA and histone modification (S. Li et al. 2016), transcriptional (Puram et al. 2017; Peng et al. 2019), and post-transcriptional regulation (Shapiro et al. 2011; Jolly et al. 2016; Pradella et al. 2017), and transcriptional noise (Han et al. 2016). Selection acts on phenotypes—not directly on genotypes—and the phenotype conferred by a genotype can be highly context-dependent. Thus, no matter the source of (epi)genetic and transcriptional diversity, it is the overall phenotypic behavior of the cell that determines its persistence and fate in a cell population. Critically important phenotypes of cancer have been categorized as “Cancer Hallmarks”: an assortment of phenotypic traits in common across nearly all cancers (Hanahan and Weinberg 2000, 2011). These hallmarks of cancer include genome instability and mutation, sustained proliferative signaling, evading growth suppressors, enabling replicative immortality, resisting cell death, inducing angiogenesis, deregulating cellular energetics, tumor-promoting inflammation, avoiding immune destruction, and activating invasion and metastasis (Hanahan and Weinberg 2011).

An instructive parallel can be drawn between the convergent evolution in cancer phenotypes towards cancer hallmarks and the phenotypic convergence observed in cave-adapted fish (Gatenby et al. 2011). The diversity of cave-adapted fish throughout the world is the result of dozens of independent evolutionary habitat transitions by lineages that span the teleost Tree of Life. Nevertheless, virtually all obligate cavefish species have converged upon similar phenotypic hallmarks that provide adaptive advantages in cave environments (Gatenby et al. 2011), a pattern of convergence that is remarkable considering these fishes span divergences that in some cases exceed the origin of mammals (Near et al. 2012). Like cavefish, many cancer types are extremely genetically diverse, but they also converge under intense selective pressure upon certain hallmarks that enable their survival.

The phenotypic convergence onto the hallmarks of cancer observed across cancer types can be associated with molecular convergence as well. Sequencing has revealed common driver mutations in the same oncogene or tumor suppressor across different cancers. Common mutations in the TP53 DNA binding domain, KRAS G12 and G13, and domains of EGFR and PIK3CA are enriched across both individual patients and multiple cancer types (Bailey et al. 2018). Convergences such as these manifest as oncogenic hotspots and tumor suppressors with high mutation loads—molecular evidence of the intense but context-dependent selective pressures on cancer cell lineages within tissues and growing tumors (Fortunato et al. 2017).

## Integrating Evolutionary Paradigms into Cancer Research

**Fig. 1.**
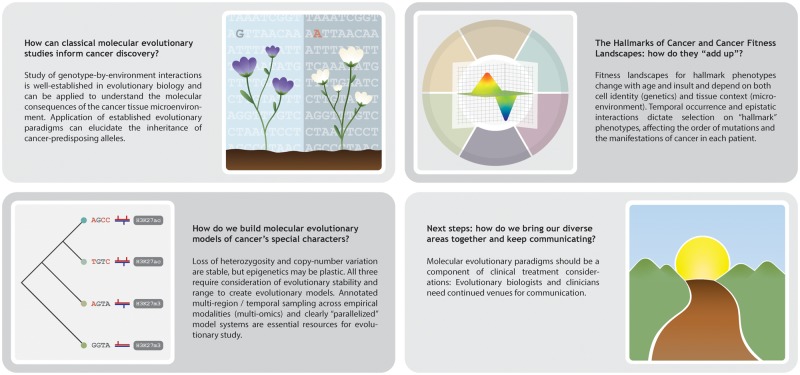
Current challenges in understanding cancer proposed by attendees at the 2019 SMBE Satellite Conference on the Molecular Biology and Evolution of Cancer.

Understanding cancer from the lens of evolutionary theory is essential to fully comprehend cancer’s behavior. Herein we present a perspective on cancer and evolution that resulted from discussion during our SMBE-sponsored satellite meeting on the molecular biology and evolution of cancer. We highlight below fields of study in which evolutionary biology and cancer research naturally intersect and present a summary of potential solutions to some of the most pressing questions related to cancer and evolution (fig. 1).

### Cross-Species Analyses of Cancer Reveals New Insights

The study of naturally-occurring cancers across species provides a unique perspective on cancer biology (Wong et al. 2019). The core clinical and molecular similarities between cancer across species have supported the longstanding use of animals with spontaneously-occurring cancers to better understand mechanistic drivers of tumors. In small animal patients, such as dogs, the similarities to humans in disease presentation, response to treatment, and the development of drug-resistance and metastasis provide an opportunity to interrogate points of therapeutic intervention and generate a thorough preclinical assessment of novel treatments.

To optimize future comparative efforts, significant energy has been placed in characterizing the genomic landscape of multiple canine cancers. Notably, while many canine cancers exhibit a similar genomic landscape to their human counterparts, novel features of the disease in dogs may also help explain some of the differences in behavior of these diseases between species. For example, recent characterization of the genomic landscape of osteosarcoma in pet dogs revealed a similar mutation burden and complex spectrum of structural aberrations to that recognized in pediatric human osteosarcoma. However, unique features of osteosarcoma in dogs, such as mutations in the epigenetic regulator, *SETD2*, and deletions in *DMD*, the gene encoding dystrophin, may help explain the more aggressive disease biology recognized in canine osteosarcoma (Perry et al. 2014; Sakthikumar et al. 2018; Gardner et al. 2019). These canine-specific molecular alterations may inform on the biology of aggressive disease or pinpoint a unique molecular subtype of aggressive human osteosarcoma. Additional examples of canine cancers with shared disease biology in people include diffuse large B-cell lymphoma and leukemias, urothelial carcinomas, and soft tissue sarcomas, among others. For example, whole-exome sequencing and RNA-sequencing of golden retrievers with hemangiosarcoma revealed similar aberrations in genes and signaling pathways (Megquier et al.). These efforts often leverage the extensive tracts of linkage disequilibrium within breeds of dogs—driven by selective inbreeding—to map molecular variants that predispose them to cancer (Sutter et al. 2004; Lindblad-Toh et al. 2005; Ostrander and Wayne 2005).

Across mammalian species, incidences of cancer are highly heterogeneous. For example, while cancer is the most common cause of death in dogs over 10 years of age, with many cancers observed at a higher incidence in dogs compared with people, other mammals, such as naked mole rats and elephants, are recognized to have a lower incidence of cancer (Tollis, Boddy, et al. 2017; Tollis, Schiffman, et al. 2017). Nevertheless, comparative investigations of cancer between species are still limited; however, emerging studies are shedding light on the mechanisms of cancer protection in some species. Investigations of elephant genomes revealed copy number gains in the tumor suppressor, *TP53*, a discovery that has since guided comparative research efforts to interrogate the role of tumor suppressor genes (Abegglen et al. 2015; Sulak et al. 2016). Additionally, animals living under protected conditions (e.g., humans, domesticated, zoo/aquarium, and laboratory animals) represent a potential boon of model systems to investigators. These animals are far more likely to reach ages where cancers are much more common and in some cases can also experience modern exposures (e.g., cigarette smoking) that enhance cancer risk (Hochberg and Noble 2017). By leveraging the unique features of cancer across multiple species, we have an unprecedented opportunity to advance future comparative and translational research efforts, thereby improving both our understanding of cancer biology and clinical outcomes for all patients.

### Phylogenetic Evolution of Tumor Progression and Metastasis

Given the fundamental importance of evolutionary paradigms in cancer, tools, and concepts designed to study evolutionary relationships (Darriba et al. 2018) are well suited to studies of cancer evolution (Somarelli et al. 2017). For example, incorporating molecular phylogenetic frameworks has led to improvements in imputation of missing base calls in single-cell sequencing data (Miura, Huuki, et al. 2018), and prediction of subclonal architecture from bulk sequencing data (Fischer et al. 2014; Miura, Gomez, et al. 2018). Studies applying low-pass whole-genome sequencing to circulating tumor DNA have demonstrated the feasibility of applying phylogenetic tools and evolutionary principles to track clonal dynamics during the evolution of chemotherapy resistance (Davidson et al. 2019). Whole-genome or whole-exome sequences can be used with slight modifications of classical methods of phylogenetic inference to reconstruct chronograms of cancer evolution (Zhao et al. 2016). Furthermore, analysis of ancestral states can be highly informative regarding the sequence of events underlying tumorigenesis, metastasis, and the evolution of resistance. Superposition of these temporally granular investigations of the molecular evolution of cancer with patient clinical information provides tremendous insight into the biological and clinical time course of cancer, yielding patient-specific cancer histories and common trajectories of specific cancer types. Continued development of tools grounded in evolutionary principles, coupled with further innovations in sequencing technologies, may help stratify patients for clinical trials and/or identify new actionable targets for therapeutic intervention. One area with intense research activity has been the estimation of clonal history (Beerenwinkel et al. 2015; Turajlic et al. 2015; Miura, Gomez, et al. 2018) and concomitant inference of selection (Williams et al. 2016, 2018; Tarabichi et al. 2018) using variant frequency data from tumor sequencing, an enterprise made especially challenging by cancer’s special molecular characteristics—clonal growth and competition, loss of heterozygosity, rampant copy number variation, and epigenetic effects. Extensive research is needed to adapt and develop molecular phylogenetic methods well suited for analyzing extensive tumor variation that can be much more complex than sequence variation in the analysis of natural populations and species.

### Leveraging Evolutionary Fitness Landscapes in Cancer

Just as fitness represents the ability of an organism to survive and create genetically related offspring, it can also represent such competitive ability for cell lineages within an individual. Recognition of evolutionary selection as a metric of cancer driver genes’ relative importance led to the calculation of scaled selection coefficients as a means of ranking the effects of cancer drivers (Cannataro, Gaffney, and Townsend 2018; Cannataro et al. 2019). However, the fitness of a phenotype conferred by these variants is determined not only just by their genotype, but also by resource availability (Yun et al. 2009; Zapata et al. 2018; Bhandari et al. 2019) and epistatic interactions (Wilkins et al. 2018; van de Haar et al. 2019). Therefore, fitness landscapes can shift when resource availability or the environment change to favor a subpopulation that is, by chance, better adapted to those new conditions. In the context of cancer, resources and environments are ever-changing. One key driver of this dynamic environment is age: inflammatory, metabolic, and mitochondrial functions change dramatically in older individuals (Davizon-Castillo et al. 2019), and mutation accumulation with age is expected to drive declines in cell renewal potential in tissues, particularly those with high turnover (Cannataro et al. 2016). These age-related changes in tissue architecture and function can alter the selective regime operating on stem or other progenitor cells. Henry et al. (2015) demonstrated that aging-associated increases in inflammation reduce the fitness of B-progenitor cells, promoting selection for progenitors with oncogenic mutations that restored their fitness, and leading to increased leukemias. As a malignancy expands, it creates additional microenvironmental hurdles that increase selection for adaptive genetic/phenotypic changes (Gatenby and Gillies 2008), some of which engender specific cancer hallmarks. Therefore, studies of gene-by-environment regulation and evolution across tissue and tumor microenvironments could form a basis for novel approaches that reduce cancer initiation and progression.

Although changing environmental conditions clearly alter tissue and tumor fitness landscapes, the phenotypic plasticity of cancer cells can also provide cells with a fitness advantage. For example, using a zebrafish metastasis model of melanoma, Heilman et al. observed that disseminated melanoma cells were unpigmented, but the metastatic colonies became differentiated and gained pigmentation once colonies were established (Gatenby and Gillies 2008; Heilmann et al. 2015). This observation is reminiscent of the epithelial–mesenchymal plasticity observed during metastatic dissemination and colonization in other solid tumors. For many epithelial-derived tumors, a subset of cells undergo a phenotypic transition from epithelial-like to mesenchymal-like. This epithelial–mesenchymal transition enables cells to migrate, invade, and disseminate; however, the increased invasive behavior as a mesenchymal-like cell comes at a cost: cells that have undergone epithelial–mesenchymal transition often slow or stop their proliferation through cell cycle arrest (Vega et al. 2004; Mejlvang et al. 2007; Hu et al. 2008). Subsequent to seeding in a new environment, though, these mesenchymal-like cells can revert back to an epithelial-like phenotype, which reawakens proliferative capacity and enables cells to colonize (Jolly et al. 2017). This phenotypic plasticity broadens the environmental conditions available to the cell and increases the cell’s overall fitness under varying resources and environments.

There are clear commonalities in fitness landscapes within and across individuals that have been demonstrated by the recurrent selection for a somewhat limited set of oncogenic mutations—particularly for the same cancer type—across many individuals. Intra-individual variability in the tissue microenvironment and phenotypic plasticity of individual cells make it challenging to discover how cancer lineages converge on fitness optima. Recurrent mutations often occur on the trunk of a clonal phylogenetic tree (Zhao et al. 2016; Yates et al. 2017), indicating strong selection for a subset of oncogenic mutations early in cancer progression. This strong selection is also indicated by the association between the prevalence of observed mutations, the pathogenicity of those mutations, and the amplitude of mutations’ functional impacts on proteins and pathways (M. Li et al. 2016). To connect prevalence to the landscape of differential fitness effects of new mutations requires accounting for the natural variability in mutation rate at all scales throughout the genome and between different tissue types (Cannataro, Gaffney, Stender, et al. 2018; Cannataro and Townsend 2018, 2019; Brown et al. 2019). The relative heights of the peaks in the fitness landscape of tumorigenesis may be leveraged in a clinical setting—as the peaks of the fitness landscape correspond to relative increases in division and survival potential of cancer cells, and thus directly inform decision making about clinical trials (Wilkins et al. 2018) and the potential for cancer cell adaptation to novel putative therapies (Cannataro, Gaffney, Stender, et al. 2018).

### Evolutionary Genomics of Cancer

Advances in sequencing technologies and analyses have continued to illuminate the dynamics of evolutionary processes in cancer. Exome sequencing revealed not only substantial inter-patient somatic genetic diversity with greater patient sampling (Robinson et al. 2015; Armenia et al. 2018; Cannataro and Townsend 2019), but also remarkable intratumoral heterogeneity (Gerlinger et al. 2012) that can be followed by disseminated metastatic diversity (Zhao et al. 2016; Reiter et al. 2019). Subsequent studies have illustrated the evolutionary dynamics at play during the emergence of therapy resistance (Gupta et al. 2017; Armstrong et al. 2019), as well as the role of nongenetic reprogramming of stromal compartments as contributors to therapy resistance (Woolston et al. 2019). For example, Mourikis et al. (2019) used machine-learning to identify a series of “helper genes” that work together with cancer driver genes to promote esophageal cancer. These helper-driver networks converged toward the perturbation of molecular processes with well-known roles in cancer, such as intracellular signaling and cell cycle progression. The perturbation of similar processes is therefore recurrent in highly heterogeneous cancers, further supporting the importance of convergent evolution in cancer.

## Discovery to Action: Adopting Evolutionary Approaches to Treat Cancer

From the selection of specific life history traits that protect organisms from cancer to the evolution of therapy-resistant and prometastatic disease states within a tumor, it is clear that the initiation, persistence, and progression of cancer is deeply rooted in molecular evolution. In exploring the connections between cancer and evolution, we asked how we can 1) use our understanding of molecular evolution to inform cancer discovery; 2) build molecular evolutionary models of cancer’s special characters; 3) better understand the relations between the hallmarks of cancer and cancer fitness landscapes; and 4) facilitate collaboration and communication between diverse areas of research (fig. 1). Potential solutions to each of these challenges highlight the need for a more expansive toolkit to integrate established evolutionary paradigms into existing cancer research activities as well as communication across evolutionary and clinical disciplines.

### Evolutionary and Ecological Paradigms Help Expand the Cancer Research Toolkit

A key concept underlying organismal evolution is the idea that environment shapes both phenotypes and the fitness values of phenotypes, leading to a fitness landscape. Likewise, cancer fitness landscapes can recapitulate and model the progression of cancer and the acquisition of its hallmarks. Application of fitness landscapes to cancer evolution requires an understanding of temporal changes in normal and cancerous tissues, in part because mutation order is a critical determinant of cancer evolution (Zhao et al. 2016; Kent and Green 2017; Gomez et al. 2018) and fitness landscapes change with age (Bilousova and DeGregori 2019; Guida et al. 2019; Nguyen et al. 2019; Rozhok and DeGregori 2019) and insult (Roper et al. 2019). Multi-regional and temporal sampling and sequencing of tumors and cells will continue to be an essential resource, enabling comprehensive monitoring of the evolutionary process underlying cancer progression. Liquid biopsies, for example, provide a noninvasive method of periodically sampling the cancer genomes within a patient, including those from tumors located in multiple regions of the body (Wan et al. 2017). Integration of longitudinal sampling with liquid biopsies, evolutionary genomics, and comparative oncology can be performed by leveraging other organisms when sampling from humans is challenging. Pet dogs acquire naturally occurring cancers; their of shorter lifespan enables time- and cost-effective data collection, and their cancers exhibit considerable biological similarity to those of their human counterparts (Schiffman and Breen 2015). At the same time, multiple model systems that can reproducibly and quantitatively demonstrate intratumoral evolution in response to treatment: patient-derived xenografts can help distinguish patterns indicating selection from stochastic evolution across such multisample studies (Kim et al. 2018). These paradigms from ecology and evolutionary biology may ultimately become essential to effective medical decision making. 

### Cross-Disciplinary Communication to Fuel Discovery and Innovation

Evolutionary paradigms are already well established for evolving populations of organisms and microorganisms. Because of the role of evolution in tumorigenesis, these paradigms are an invaluable resource for application to the better understanding of cancer origination, development, and biology. For example, metastasis can be studied through the lens of movement ecology, which describes how external pressures in an organism’s environment, combined with the organism’s intrinsic motivations and abilities, ultimately influence migration (Amend et al. 2016). Fitness of neoplastic cells can be understood within the framework of life history theory, which suggests that limited resources necessitate tradeoffs in energy allocation to functions such as growth, maintenance, and reproduction (Boddy et al. 2018). Conceptual parallels between organismal and cancer evolution suggest that phylogenetic methods and tools can be adapted to study cancer from a genetic and ecological perspective; cancer can likewise be exploited as a molecular model to better understand fundamental evolutionary paradigms. Increased connection and communication between evolutionary ecologists, cancer biologists, and clinicians has enormous potential to make a positive impact on our understanding of cancer and ultimately reveal novel approaches to help prolong and improve the lives of cancer patients. 

## Acknowledgment

This Perspective was developed with the assistance of funding from the Society of Molecular Biology and Evolution that supported the SMBE Satellite Meeting on the Molecular Biology and Evolution of Cancer at the Yale School of Public Health, April 12–13, 2019, organized by J.S. and J.P.T. Additional support was provided by the Notsew Orm Sands Foundation to J.P.T. and by a grant from the National Institutes of Health to S.K. (LM012487).
